# Effect of Machining-Induced Subsurface Defects on Dislocation Evolution and Mechanical Properties of Materials via Nano-indentation

**DOI:** 10.1186/s11671-019-3212-7

**Published:** 2019-12-09

**Authors:** Quanlong Wang, Meiping Wu, Chaofeng Zhang, Yanming Lv, Xiaogang Ji

**Affiliations:** 1 School of Mechanical Engineering, Jiangnan University, Wuxi, 214122 People’s Republic of China; 2 Jiangsu Key Laboratory of Advanced Food Manufacturing Equipment &Technology, Wuxi, 214122 People’s Republic of China

**Keywords:** Subsurface defects, Dislocation evolution, Mechanical properties, Nano-indentation, Molecular dynamics simulation

## Abstract

Subsurface defects have a significant impact on the precision and performance of nano-structures. In this paper, molecular dynamics simulation of nano-indentation is performed to investigate the effect of machining-induced subsurface defects on dislocation evolution and mechanical properties of materials, in which the specimen model with subsurface defects is constructed by nano-cutting conforming to reality. The formation mechanism of subsurface defects and the interaction mechanism between machine-induced defects and dislocation evolution are discussed. The hardness and Young’s elastic modulus of single crystal copper specimens are calculated. The simulation results indicate that there exist stable defect structure residues in the subsurface of workpiece, such as atomic clusters, stacking fault tetrahedral, and stair-rod dislocations. Secondary processing of nano-indentation can restore internal defects of the workpiece, but the subsurface damage in the secondary processing area is aggravated. The nano-indentation hardness of specimens increases with the introduction of subsurface defects, which results in the formation of work-hardening effect. The existence of subsurface defects can weaken the ability of material to resist elastic deformation, in which the mutual evolution between dislocations and subsurface defects plays an important role.

## Background

Ultra-precision fabrication at nano-metric scale is widely considered to be an effective method to obtain nano-components with submicron dimensional accuracy and nanoscale surface quality [1]. Some stable subsurface defects are left inside the workpiece after fabrication [2–5]. The subsurface defects not only solely affect the processing accuracy and surface quality, but also critically affect the mechanical properties and service life of nano-components. Many studies about subsurface defects have been carried out by molecular dynamics (MD) method, mainly focusing on the formation and evolution of subsurface defect s[6, 7], the thickness of subsurface defects (SSD) layer [8, 9], and the influence subsurface defects on surface integrity [10, 11]. However, the effect of subsurface defects on mechanical properties of workpiece materials is less studied. The mechanical properties of nano-structures are critical to their service performance and life. Therefore, the effect of subsurface defects on mechanical properties of materials has become the key issues to be investigated.

Many researches have been conducted to deliberate the SSD layer by molecular dynamics simulation of nano-cutting process. Narayanan [12] studied the formation of stacking fault tetrahedral (SFT) in single crystal gold and introduced deformation-induced mechanism of SFT. Inamura [13] explored the chip formation and material slip deformation during nano-cutting process and pointed out that the chip formation is mainly induced by shear-slip deformation. Pei [14] studied the effect of cutting parameters on dislocation evolution and cutting forced during nano-cutting process and found that when the workpiece is larger than 40 nm, the size effect is not significant. Dai [15] and Liu [16] adopted MD simulation and experiment methods respectively to study the influence of diamond tool structure and size effect on the evolution of workpiece subsurface defects. The previous studies show that there exist stable subsurface defects in the workpiece after nano-cutting. Cutting parameters and tool geometry parameters have great influences on the thickness and evolution of subsurface damage layer, and even on the processing accuracy. However, the mechanical properties of workpiece materials cannot be calculated by analyzing the relevant data obtained by nano-cutting.

Nano-indentation is an effective technique to characterize the mechanical properties such as hardness and elastic modulus [17]. A lot of studies on nano-indentation have been carried out to evaluate the performance of mechanical properties by experimental and theoretical models. Zimmerman [18] analyzed the dislocation emission in nano-indentation process by the slip vector. Ruestes [19] studied nano-indentation of single crystal Fe by MD simulation and found that dislocation generation in subsurface is necessary to remove the material from the indentation zone. Huang [20] performed MD simulations of nano-indentation on single crystal diamond matrix and found that the deformation of diamond material under indentation was dominated by the nucleation and propagation of 〈110〉 {111} dislocation loops. Sharma [21] constructed a hard particle model artificially in copper matrix and analyzed the influence of hard particle on subsurface defects evolution in machining process. Peng [22] investigated the strengthening mechanisms of graphene coatings on Cu substrate by nano-indentation, which is resulted from the stress homogenization effect generated by the interface. From the above analysis, it can be seen that the previous studies on the effect of subsurface defects are mainly based on perfect crystal materials or artificial constructing hypothetical defects, which is far away the actual subsurface defects. Therefore, constructing the workpiece model with subsurface defects in realistic is essential to analyze the influence of subsurface defects on mechanical properties of workpiece.

In this paper, nano-cutting method was adopted to obtain the workpiece model with subsurface defects conforming to practical characteristics. On this basis, nano-indentation simulation was carried out to study the effect of subsurface defects on the mechanical properties of single crystal copper. Firstly, the formation and evolution mechanism of subsurface defects during nano-cutting process will be discussed, and the typical defect structures of workpiece subsurface after nano-cutting will be analyzed. Secondly, the interaction mechanism between machining- induced subsurface defects and dislocation nucleation during indentation will be analyzed. Thirdly, based on the load-displacement data obtained by nano-indentation, the hardness and Young’s elastic modulus of single crystal copper specimens will be calculated. Finally, some novel conclusions will be summarized.

## Methods

### Simulation Model

**Fig. 1:**
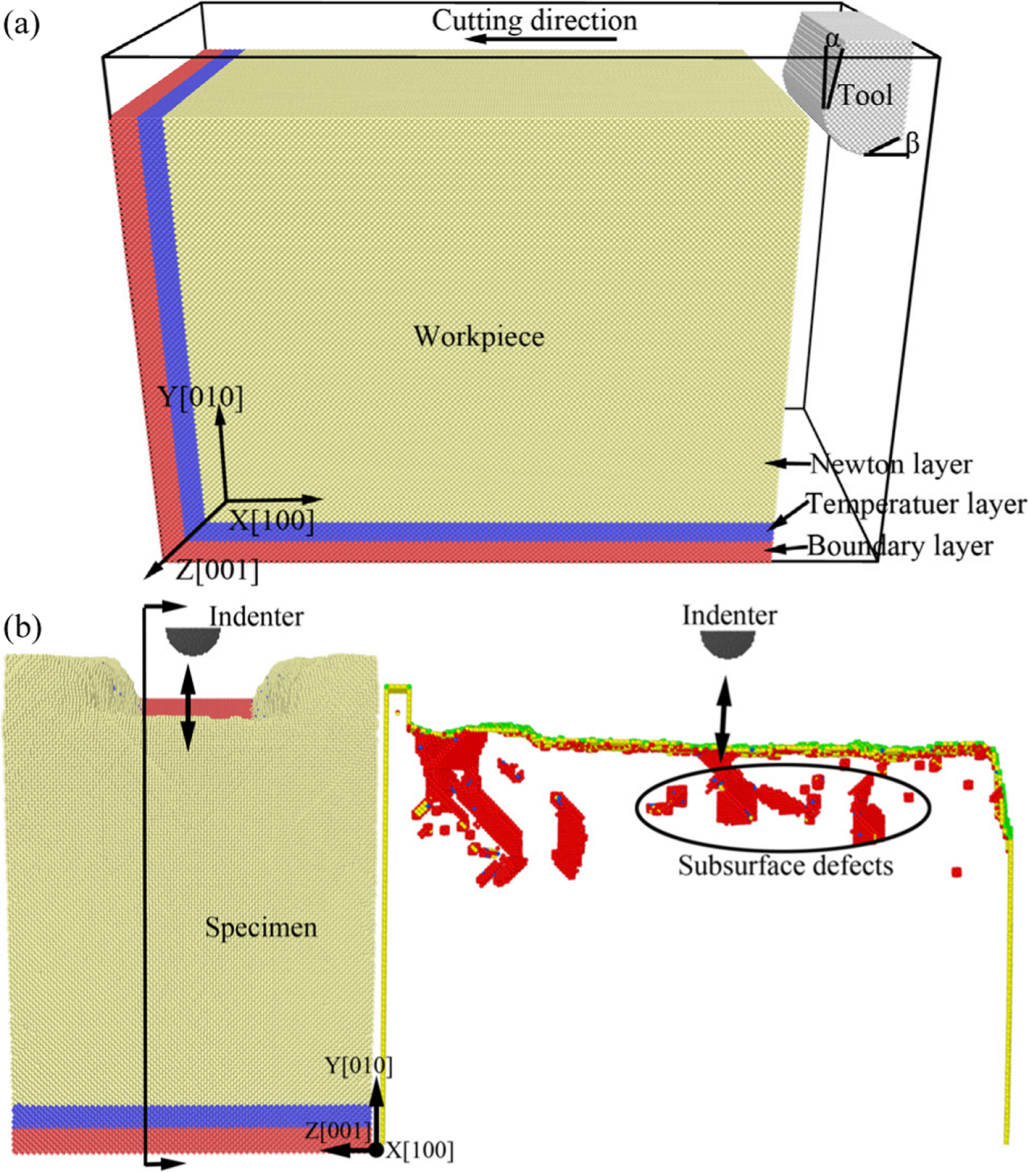
Schematic diagram of 3-D MD simulation models of single crystal copper for nano-cutting and nano-indentation. **a** The model for nano-cutting process. **b** The model for nano-indentation process with subsurface defects

In order to investigate the effect of subsurface defects on mechanical properties of materials in nano-machining, the specimen model with subsurface defects should be constructed. In this research, it is realized by MD simulation of nano-cutting process. First of all, the MD simulation model is established and the nano-cutting process simulation is performed. *Then the specimen and cutting tool relaxed for sufficient time during MD simulation*. Finally, some stable *defects remained in subsurface* of workpiece. The schematic diagram of the three-dimensional MD simulation models is shown in Fig. 1, in which the nano-cutting model is shown in Fig. 1a and the nano-indentation model with subsurface defects is shown in Fig. 1b. In Fig. 1, the materials of workpiece and specimen are single crystal copper and the tool and indenter are diamond materials. The arc blade diamond tool is used in nano-cutting process, and the tool edge radius is 3 nm. The indenter is hemispheric shape in nano-indentation process, and the diameter is 6 nm. The workpiece and specimen are divided into three parts, which are Newton layer, temperature layer, and boundary layer, respectively. In order to reduce the size effect and boundary effect, the periodic boundary condition (PBC) is adopted at [010] direction of the simulation system. To avoid the initial interaction between tool and workpiece, the cutting tool is put 3 nm top right of the workpiece and the indenter is put 6 nm up to the specimen. The detailed simulation parameters are shown in Table 1.

**Table 1:** MD simulation conditions in 3-D nano-machining

Machining parameters	Value
Potential function	Tersoff, Morse, EAM
Workpiece (specimen)	Single crystal copper
Tool (indenter)	Diamond
Lattice structure	FCC
Workpiece/specimen size	40 nm × 30 nm × 22 nm
Tool rake angle *α*	15°
Tool clearance angle *β*	8°
Tool edge radius *r*	3.0 nm
Machining direction	(100) [100], (100) [010]
Cutting depth	3 nm
Cutting speed	100 m/s
Indenter diameter *d*	6.0 nm
Indentation depth	2~4 nm
Indentation speed	50 m/s
Timestep	1 fs

### Interatomic Potential Functions

In this research, the three-dimensional MD simulations were performed by large-scale atomic/molecular massively parallel simulator (LAMMPS). The parallel computation was realized under the help of message passing interface library. The Morse potential, Embedded-atom Method (EAM) potential, and Tersoff potential are used in the simulation, which are invoked from LAMMPS software package. The interaction between Cu atoms in workpiece and C atoms in the tool is calculated by Morse potential which is shown in Eq. 1 [23].

1 $$ u\left({r}_{ij}\right)=D\left[\exp \left(-2\alpha \left({r}_{ij}-{r}_0\right)\right)-2\exp \left(-\alpha \left({r}_{ij}-{r}_0\right)\right)\right] $$

where *r*_0_, *α*, and *D* respectively are atomic spacing, elasticity modulus, and binding energy. The value is shown in Table 2.

**Table 2:** Parameters value in Morse potential

*r*_0_ (*Ả*)	α (*Ả*^−1^)	*D* (eV)
2.05	5.140	0.087

The interatomic function among Cu atoms in workpiece is described by EAM potential which is shown in Eqs. 2, 3 [24, 25].

2 $$ E=\sum \limits_i^N\left[F\left({\rho}_i\right)+\sum \limits_{j>i}^Nu\left({r}_{ij}\right)\right] $$

3 $$ {\rho}_i=\sum \limits_jf\left({r}_{ij}\right) $$

The interaction between carbon atoms in diamond tool is calculated by Tersoff potential which is shown in Eqs. 4, 5 [26].

4 $$ E=\frac{1}{2}\sum \limits_{i\ne j}{V}_{ij} $$

5 $$ {V}_{ij}={f}_c\left({r}_{ij}\right)\left[{V}_R^{\hbox{'}}\left({r}_{ij}\right)+{b}_{ij}{V}_A\left({r}_{ij}\right)\right] $$

where *f*_*c*_(*r*_*ij*_) is truncation function between atoms, *V*_*A*_(*r*_*ij*_) is the dual potential of absorption term, *V*_*R*_(*r*_*ij*_) is the dual potential of repulsion term, and *r*_*ij*_ is atomic distance between atom *i* and atom *j*.

### Defect Analysis Methods

In nano-cutting of single crystal copper, deformation and dislocations are nucleated at subsurface of the workpiece. In this paper, the centro-symmetry parameter (CSP) is introduced to analyze the dislocation nucleation and defect evolution of workpiece. For face center cubic (FCC) materials, the CSP value can be calculated by Eq. 6 [27].

6 $$ CSP=\sum \limits_{i=1}^6{\left|{R}_i+{R}_{i+6}\right|}^2 $$

where *R*_*i*_ is the same length neighboring atoms and *R*_*i+6*_ is the opposite direction neighbor atoms. The CSP values of FCC crystal, partial dislocation, stacking fault, and surface atoms are 0, 2.1, 8.3, and 24.9, respectively [27].

The CSP method is able to identify the atomic staggered, but cannot recognize the local atomic crystal structure state of workpiece. Therefore, the common neighbor analysis (CNA) is introduced to identify the local crystal structure defect. In the original CNA method, proposed by Honeycutt and Andersen [28], the various structures are represented by diagrams. Currently, there are five kinds of CNA patterns in OVITO [29], where the local crystal structures are identified as face center cubic (FCC), close-packed hexagonal (HCP), body centered cubic (BCC), icosohedral (ICO), and unknown, respectively. In this paper, the dislocation extract algorithm (DXA) [30] is also introduced to analyze the evolution of dislocation defect. By DXA, the different crystal structures in workpiece will be marked with different colors and the dislocation defects in the workpiece will be represented by lines of different colors.

## Results and Discussion

### Subsurface Defect Evolution in Nano-cutting Process

**Fig. 2:**
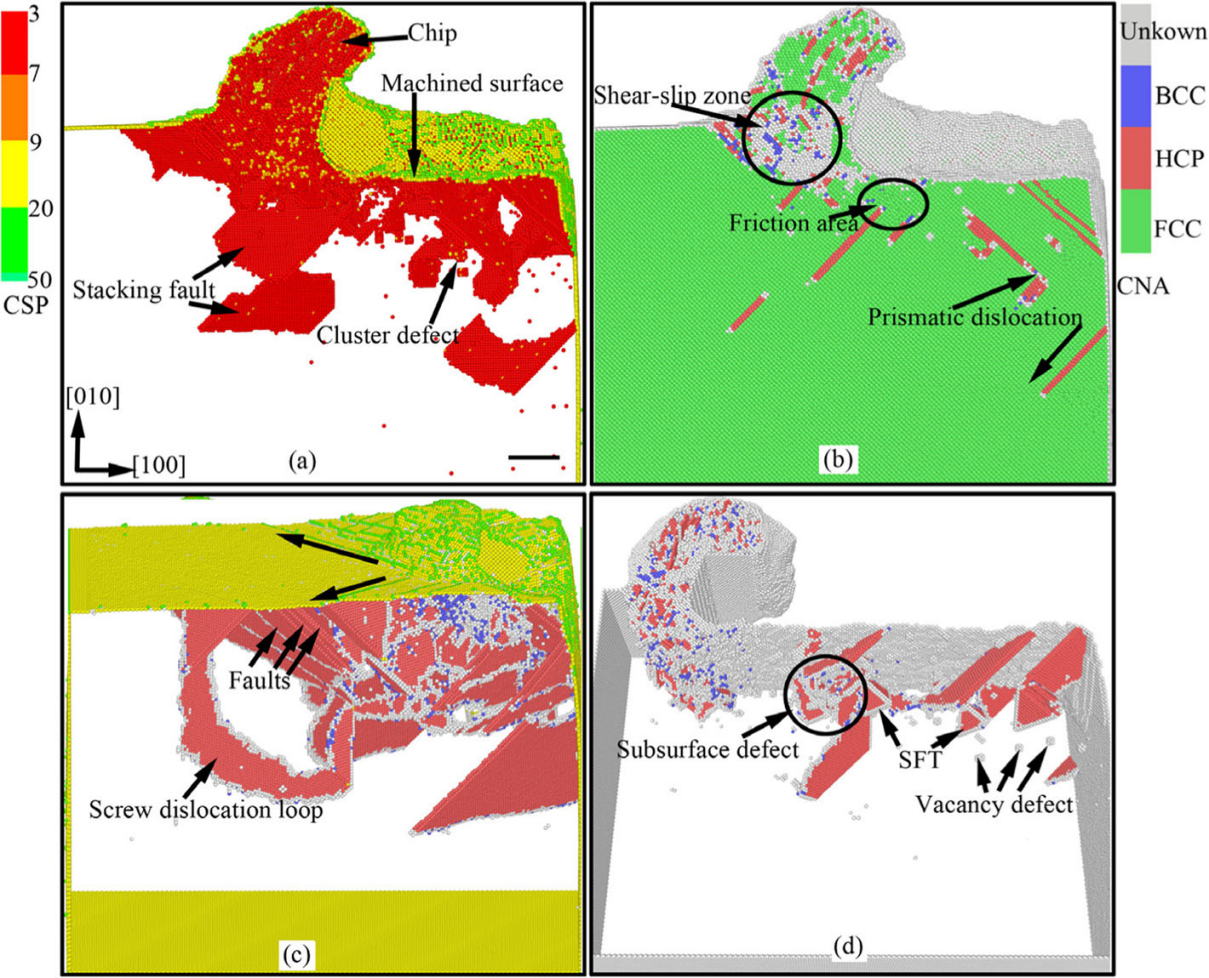
Instant views of the subsurface defect evolution during nano-cutting process of single crystal copper (Color online, scale bar 5 nm). The cutting distances of **a**, **b**, **c**, and **d** respectively are 18 nm, 18 nm, 6 nm, and 32 nm

In nano-cutting process, under the extrusion and shearing action of cutting tool on workpiece, the workpiece surface material with the thickness of cutting depth is removed, and a new machined surface with a certain size accuracy and surface quality is formed. The complex elastic-plastic deformation including stress deformation and thermal deformation is occurred in surface and subsurface of workpiece accompanying the energy transformation and the stress concentration. Therefore, the subsurface damage layer is formed and the chip is removed. Figure 2 shows the instant views of the subsurface defect evolution and the material removal during nano-cutting process of single crystal copper. In Fig. 2, the atoms are colored by the value of the CSP and CNA analysis results. In Fig. 2a and c, the yellow, green, red, and blue atoms are surface atoms, surface defects atoms, subsurface defect atoms, and FCC atoms. It can be seen from Fig. 2 that a plenty of typical defect structures are formed in subsurface of the workpiece during nano-cutting process, such as point defects, vacancy defects, stacking faults, cluster defects, prismatic dislocations, and screw dislocation loop.

Due to the squeezing and shearing effect of the cutting tool, the shear-slip deformation is generated for the atoms in front of the rake face and the primary shear-slip zone is formed in workpiece during the nano-cutting process, as shown in Fig. 2b. Some of these atoms slip up along the rake face and the cutting chip is formed as shown in Fig. 2a. Some move downward and the machined surface is formed under the squeezing friction of the tool flank face as shown in Fig. 2b. Others move inward and the subsurface defects are formed, such as stacking faults, cluster defects, and prismatic dislocations, shown in Fig. 2a and b.

On account of the friction and extrusion of tool flank surface, the energy accumulation is occurred for atoms near flank face and the atoms are become into high-energy atoms. When the atomic energy is surpassed a certain level, the energy carried by energetic atoms will be released and the dislocation is formed under the energetic atoms driving. Therefore, plenty of dislocations are formed in flank face friction area, shown in Fig. 2d. With the machined surface being formed, dislocations nucleate, extend, and annihilate, at subsurface. Finally, the defect structures such as stacking faults, SFT, and vacancy defects are left in subsurface, as indicated in Fig. 2d. The stacking faults are nucleated at shear-slip zone below the cutting tool, then extended into the workpiece, and finally annihilated at free surface of workpiece. Eventually, the dislocation line is formed at workpiece surface. And the dislocation line is extended along $\left[\overline{1}0\overline{1}\right]$, $\left[\overline{1}01\right]$, and [101] directions. The screw dislocation loop, which is located at the edge of the shear-slip zone, is consisted of several stacking faults and a series of screw dislocations. The screw dislocation is formed under the driving of the compression stress state of shear-slip zone [11].

**Fig. 3:**
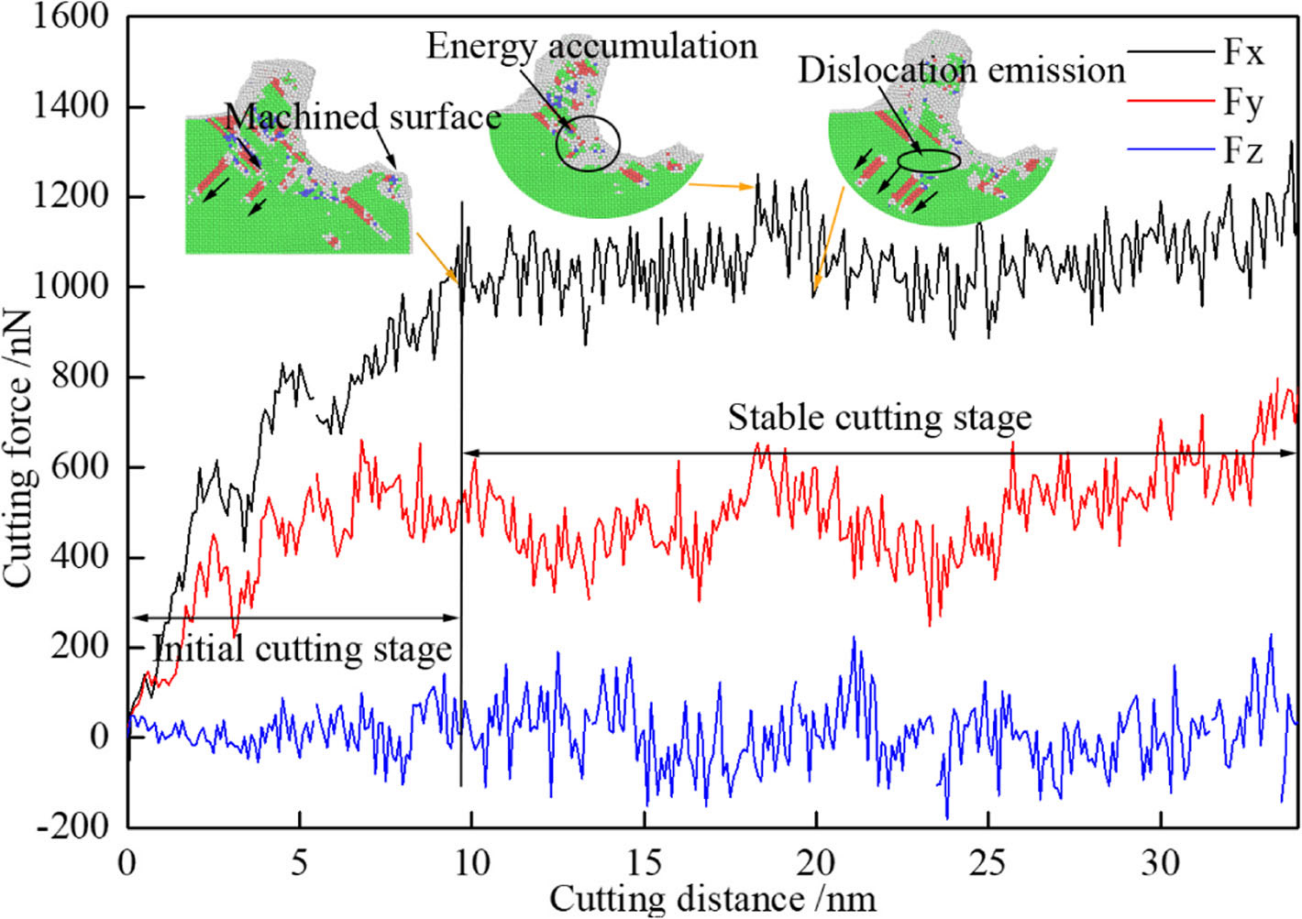
The variation curve of cutting force with the cutting distance (color online). The black, red, and blue curves respectively are the feed force (Fx), the back force (Fy), and the tangential force (Fz)

In nano-cutting process, the dislocations are nucleated and extended under the action of the cutting tool. Accompanying with the aggregation and release of energy, the cutting force is fluctuated with the increase of cutting distance, which is shown in Fig. 3 in three dimensions. In Fig. 3, the black, red, and blue curves respectively are the feed force (Fx), the back force (Fy), and the tangential force (Fz). It can be seen from Fig. 3 that the cutting process is divided into two periods which are initial cutting stage and stable cutting stage. During the initial cutting stage, the feed force and the back force have a rapidly increase, rectilinearly. The maximum value of the feed force is reached more than 1100 nN, but the back force is just arrived around 600 nN. When the tool tip cuts into the workpiece completely, the machined surface is formed, shown as the first small graph signed as Machined surface in Fig. 3. Then, the nano-cutting process is transferred to stable cutting stage. At stable cutting stage, all the three forces are fluctuating at their equilibrium positions. The average feed force is at about 1000 nN, and the average back force is only around 500 nN. It can be seen from Fig. 3 that the feed force has a rapidly decrease at the cutting distance of 20 nm. It is because that the energy accumulation is arrived at a certain level which is the critical lattice strain energy, shown as the second small graph in Fig. 3. Meantime, a big resistance is acted on the cutting tool which results in the cutting force being a peak value. Then, the energy is released which results in the dislocation emission, and the cutting force is decreased, shown as the third small graph in Fig. 3. Therefore, the cutting force is fluctuated during the stable cutting stage. The nucleation, extension, and annihilation of dislocations led to the fluctuation of the cutting force and result in the subsurface defect existing in the workpiece, finally.

**Fig. 4:**
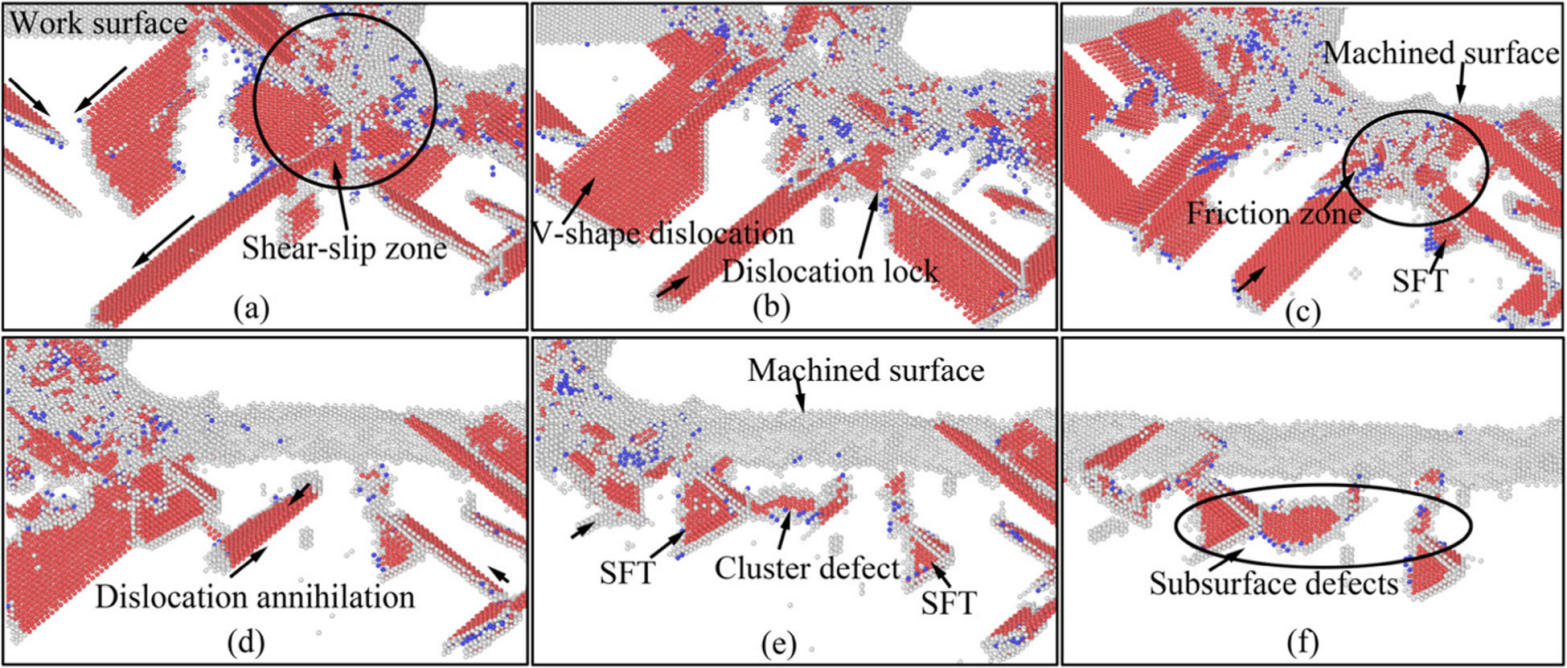
Subsurface defects evolution of workpiece (color online). The cutting distances of **a**, **b**, **c**, **d**, **e**, and **f** respectively are 8 nm, 10 nm, 12 nm, 20 nm, 24 nm, and 32 nm

In order to detailed investigate the evolution and emotion of dislocation defects in subsurface of workpiece during nano-cutting process, the dislocation distribution and its variation with the cutting distance are minutely analyzed by CNA method. The subsurface defect evolution of workpiece at a certain area is shown in Fig. 4, in which the cutting distances of Fig. 4a, b, c, d, e, and f respectively are 8 nm, 10 nm, 12 nm, 20 nm, 24 nm, and 32 nm. It can be seen from Fig. 4a that a lot of dislocation defects are nucleated at shear-slip zone under the extrusion shearing action of cutting tool during preliminary stage of cutting process. Particularly, a V-shaped dislocation and a fault are formed under the driving action of atomic stress and energy in the shear-slip zone, shown in Fig. 4b. In the subsequent processing process, the shear-slip zone is moved forward with the cutting tool keep moving. Due to the deformation energy decreasing, the fault is gradually annihilated. During the cutting tool keep moving forward, the flank face friction zone is moved near the fault. And the roughness machined surface is formed under the extrusion and friction of the flank face. Then the fault is continued annihilating and being gradually detached from the workpiece surface, as shown in Fig. 4d. Finally, the fault is transformed into cluster defect which is steadily existed in subsurface of workpiece. Similarly, the formed V-shaped dislocation is gradually evolved into SFT under the interaction of two stacking faults and a dislocation lock. These stable defects are composed the subsurface deformed layer together, as shown in Fig. 4e and Fig. 4f.

**Fig. 5:**
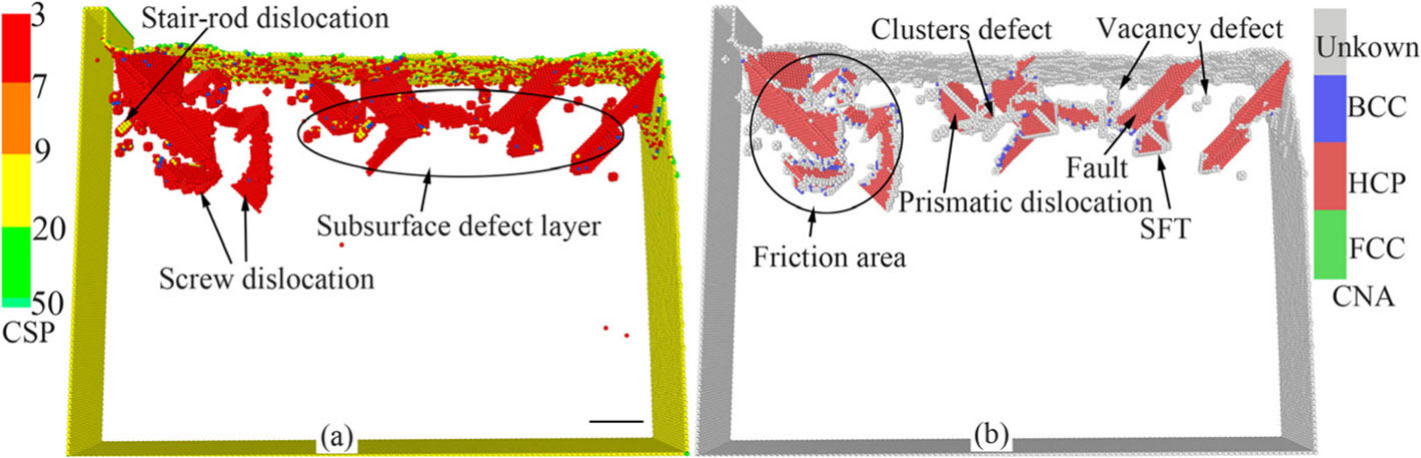
Distribution of residual defects in subsurface of workpiece after nanometer cutting (color online, scale bar 5 nm). **a** The yellow, green, red, and blue atoms are surface atoms, surface defects atoms, subsurface defect atoms, and FCC atoms. **b** The green, red, gray, and blue atoms are FCC, HCP, unknown, and BCC structure

As we known, the residual stress release and *internal defect recovery will occur* on the workpiece after aging treatment. In actual nano-fabrication, some of the subsurface defects formed during processing will be disappeared after machining process. In order to simulate the state of the workpiece after aging treatment, molecular dynamics relaxation is performed on the cutting system for a long time. The distribution diagram of residual defect in subsurface of workpiece after MD relaxation for a long time is shown in Fig. 5, in which the atoms are colored according to the analysis results by CSP and CNA methods. It can be seen from Fig. 5 that the primary dislocations are annihilated after relaxation. The vacancy defects, stacking faults, atomic cluster, prismatic dislocation, screw dislocation, SFT, and stair-rod dislocation are existed in subsurface of workpiece, as shown in Fig. 5. According to the analysis above, these defects, which are formed under the induction of complex internal stress and the interaction of dislocation defects, will affect the size accuracy and surface quality of the workpiece after nano-processing.

### Nano-indentation Test on Copper Specimen with Machining-Induced Subsurface Defects

**Fig. 6:**
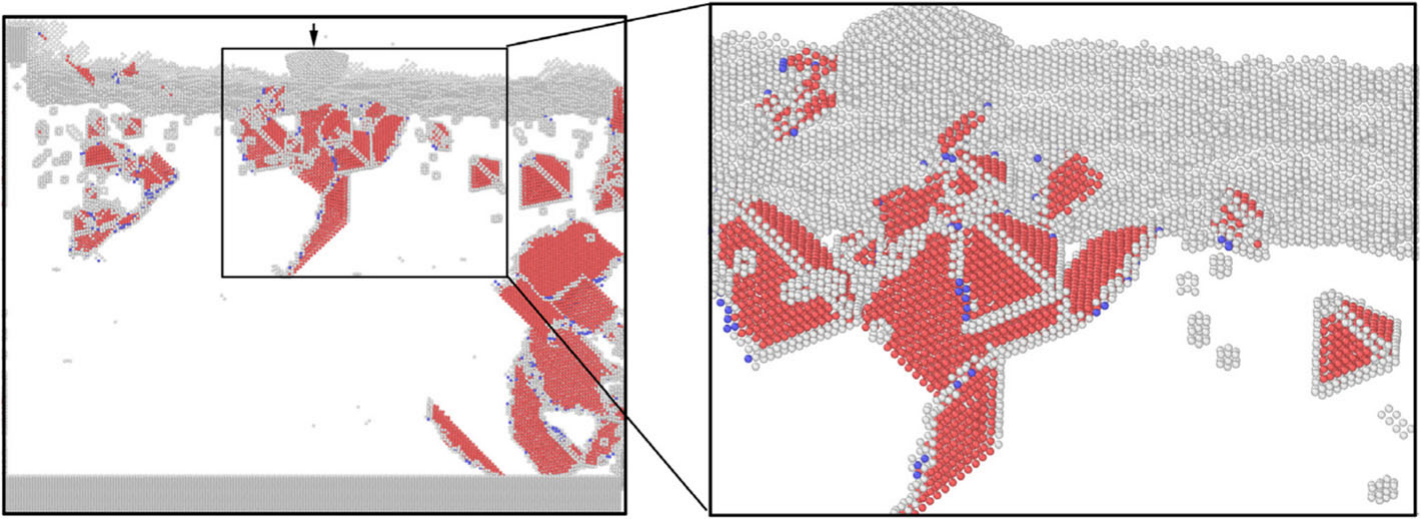
Subsurface defects distribution of workpiece in initial indentation state (color online). The red, gray, and blue atoms are HCP, unknown, and BCC structure

**Fig. 7:**
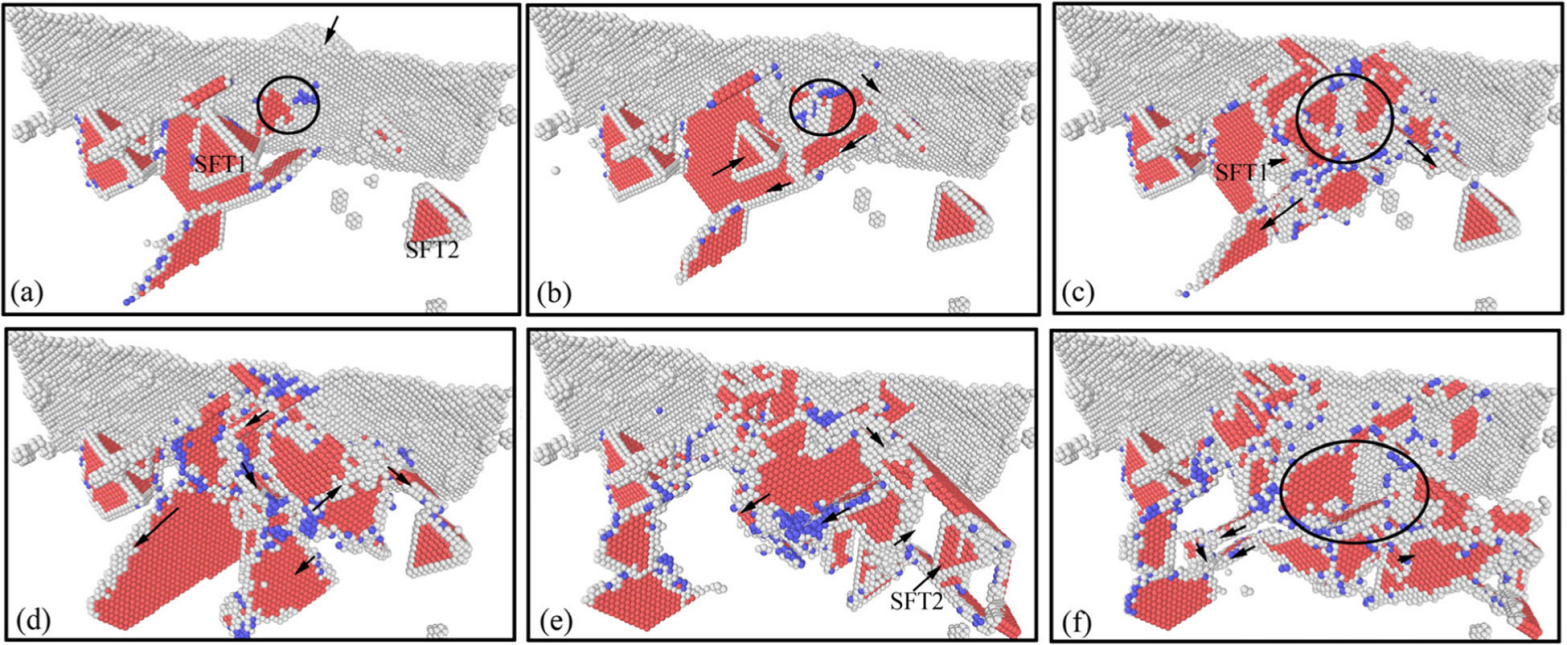
Subsurface dislocation defects evolution in loading process of nano-indentation (color online). The corresponding indentation depths of **a**–**f** are 0 nm, 0.5 nm, 1 nm, 2 nm, 3 nm, and 4 nm, respectively

**Fig. 8:**
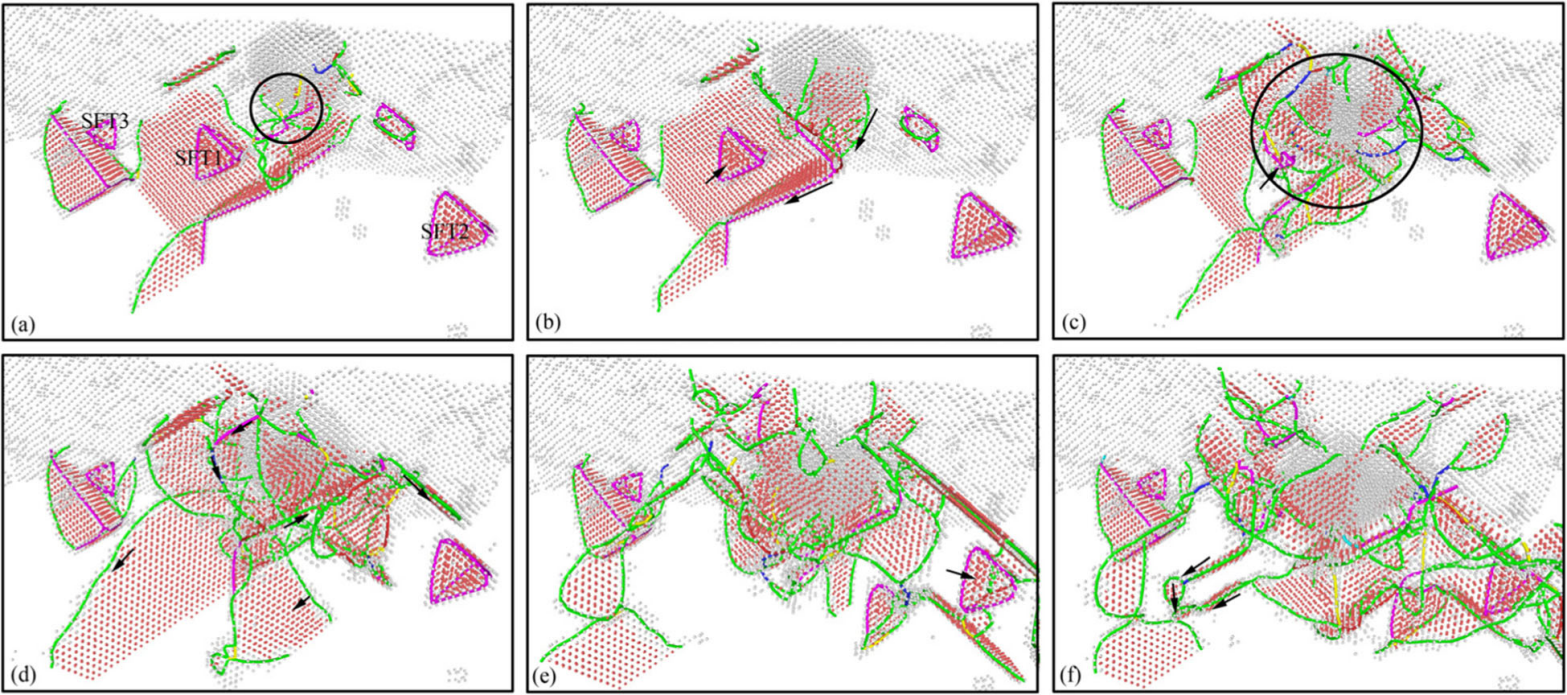
Distribution of subsurface dislocations in loading process of nano-indentation (color online). The corresponding indentation depths of **a**–**f** are 0 nm, 0.5 nm, 1 nm, 2 nm, 3 nm, and 4 nm, respectively. Color scheme: deep blue for perfect dislocations, green for Shockley dislocations, pink for Stair-rod dislocations, yellow for Hirth dislocations, light blue for Frank dislocations, and red for unidentified dislocations

**Fig. 9:**
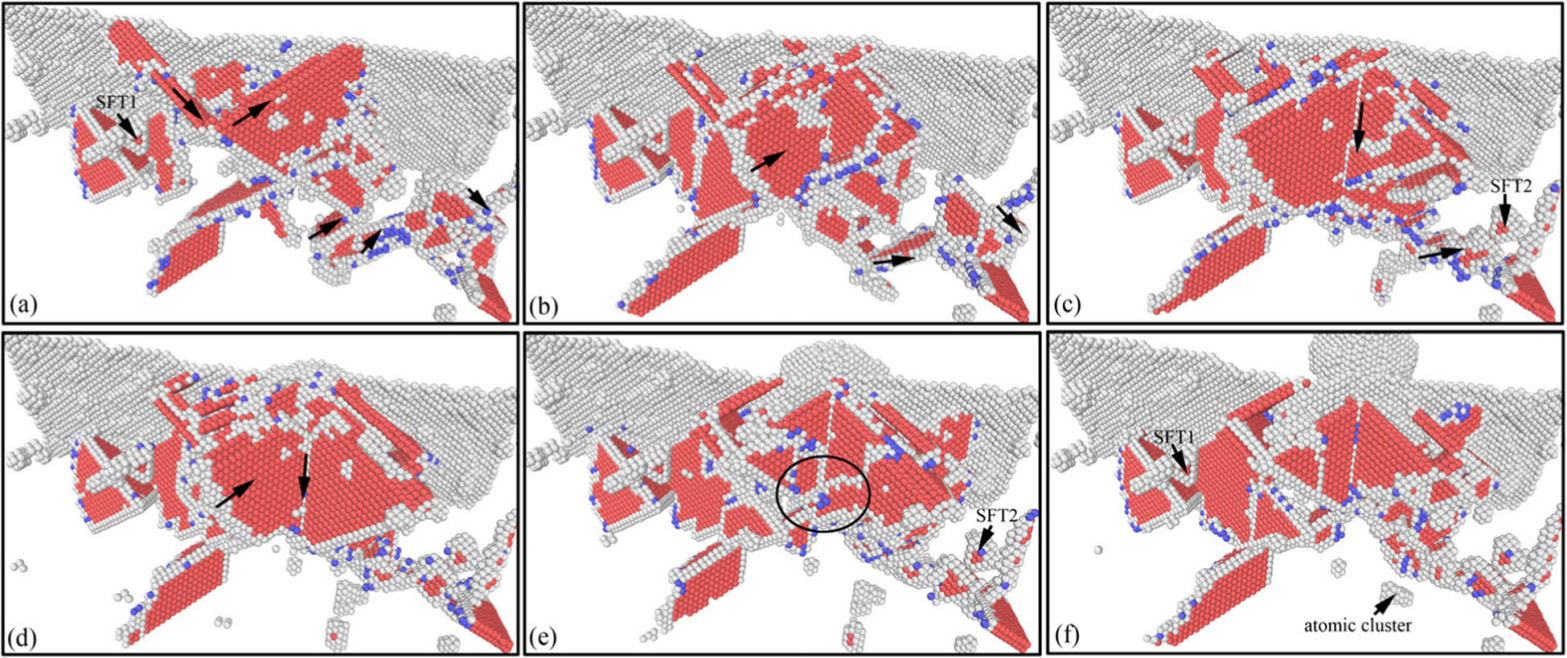
Subsurface defects evolution in unloading process of nano-indentation (color online). The corresponding indentation depths of **a**–f are 4 nm, 3 nm, 2 nm, 1 nm, 0 nm, and − 1 nm, respectively

**Fig. 10:**
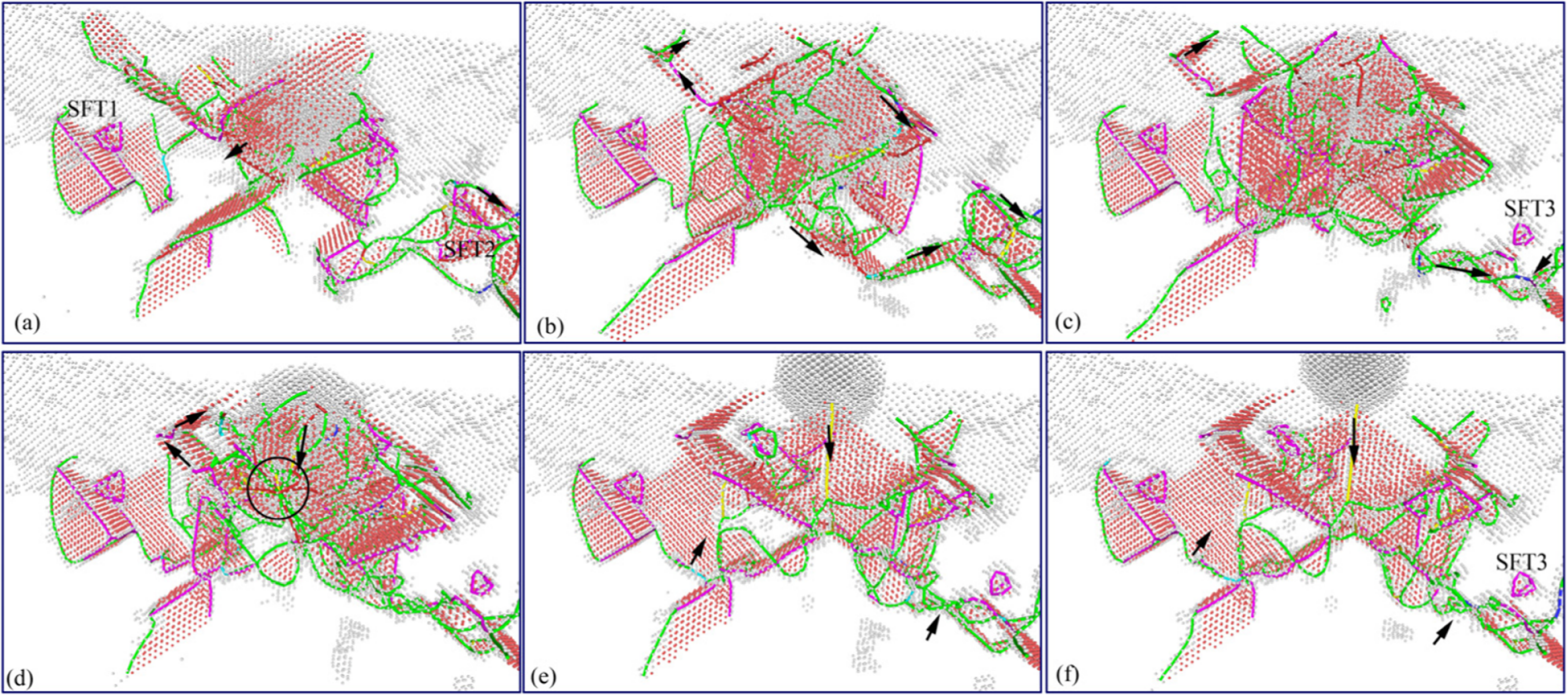
Distribution of subsurface dislocations in unloading process of nano-indentation (color online). The corresponding indentation depths of **a**–**f** are 4 nm, 3 nm, 2 nm, 1 nm, 0 nm, and − 1 nm, respectively. Color scheme: deep blue for perfect dislocations, green for Shockley dislocations, pink for Stair-rod dislocations, yellow for Hirth dislocations, light blue for Frank dislocations, and red for unidentified dislocations

The machining-induced subsurface residual defects govern the mechanical properties of the surface, especially the hardness and Young’s modulus. Therefore, the investigation of the nucleation and interaction of dislocations during nano-indentation seems strongly necessary. In order to investigate the influence of the machining-induced subsurface defects on the mechanical properties of single crystal copper, the nano-indentation process on the specimen after nano-cutting was put into practice with the same parameters as nano-cutting simulation. The simulation result is shown in Figs. 6, 7, 8, 9 and 10. The instantaneous atomic image of subsurface defect distribution in the initial state of nano-indentation is shown in Fig. 6. It can be seen from the figure that there are several SFTs, a V-shaped dislocation, some prismatic dislocations, and some cluster defects below the indenter. These subsurface defects can affect the dislocation nucleation and expansion of the workpiece during the nano-indentation process. And then the mechanical properties of the workpiece material are changed.

The atomic evolution image of subsurface dislocations during nano-indentation loading process is shown in Fig. 7. It can be seen from Fig. 7a that the initial nucleation of dislocation is occurred on the surface of the specimen when the indenter contacts the surface of the specimen. With the downward pressure of the indenter, the nucleated dislocations are expanded gradually. Meanwhile, under the influence of the stress exerted by the indenter, a large number of dislocations nucleated and moved along the slip system, as shown in Fig. 7b. Under the interaction between newly formed dislocations and original subsurface defects, some simple defects are disappeared gradually, and the V-shaped dislocations continue to be evolved and annihilated gradually. The SFT under the indenter is decreased gradually, as shown in Fig. 7c. As the indentation process going ahead, the scale of new nucleated dislocation defects increases, and the V-shaped dislocation and SFT1 under indenter are disappeared gradually. Simultaneously, the dislocation defects formed during indentation gradually are evolved into prismatic dislocation loop, in which stacking faults are gradually disappeared, as shown in Fig. 7d. The newly formed prismatic dislocation loop continues extending to SFT2 at the lower right of the indenter. Because of its stable structure, the SFT2 remain undeformed during the expansion of the prismatic dislocation loop, as shown in Fig. 7e. As the indenter pressing down, the prismatic dislocation loop continues expanding downward, and the scale of the dislocation defects in the subsurface area is increased gradually. The SFT2 structure is stably existed in the subsurface of the specimen and has no change during the movement and interference of the dislocation defects, as shown in Fig. 7 f.

In order to show the evolution and distribution of subsurface defects during indentation process more clearly, the DXA method is used to analyze the specimen after indentation. The image of subsurface dislocation distribution at each time corresponding Fig. 7 is obtained by DXA analysis, as shown in Fig. 8. Dislocations are colored based on following scheme: deep blue for perfect dislocations, green for Shockley dislocations, pink for Stair-rod dislocations, yellow for Hirth dislocations, light blue for Frank dislocations, and red for unidentified dislocations.

It can be seen from Fig. 8 that the workpiece subsurface mainly consists of Shockley dislocation and Stair-rod dislocation in the initial indentation state. The SFT existed in subsurface is a regular tetrahedral structure consisting of six stair-rod dislocations. When the indenter contacts the workpiece, the dislocation nucleation on the workpiece surface is yellow Hirsh dislocations, as shown in Fig. 8a. As the indenter pressing down, a large number of dislocations are nucleated and moved along the slip system. The yellow Hirsh dislocations are gradually changed to green Shockley dislocations, as shown in Fig. 8b. Under the interaction between newly formed dislocations and the original SFT1, some simple dislocations are disappeared, gradually, as shown in Fig. 8c. The scale of subsurface dislocations increases with the indenter dropping down, and the newly formed dislocations are mainly green Shackley dislocations. The interaction between Shockley dislocations and SFT1 results in the size of the SFT1 gradual decrease and eventual disappearance of SFT1, as shown in Fig. 8d. With the increase of defects’ scale, the number and types of dislocations in subsurface of the specimen increase. There are red unknown types of dislocations formed in subsurface, and several Shockley dislocations constituted the prism dislocation loop, as shown in Fig. 8e. Under the interaction of the Shockley dislocation and the Stair-rod dislocation, the SFT2 and SFT3 far from the indentation region did not disappear at last, due to the weaker extrusion of the indenter, as shown in Fig. 8f.

The evolution image of subsurface dislocation during nano-indentation unloading process is shown in Fig. 9. Figure 10 shows the distribution image of subsurface dislocation corresponding in Fig. 9. From the two graphs, it can be seen that the scale of subsurface defects increases at first, and then gradually decreases during the process of the indenter upward moving. It is caused by the comprehensive function between continuous release of the material deformation energy and the adhesion force exerted by the indenter on the specimen. In the initial stage of unloading process, the upward adsorption force of the indenter effect on the specimen is not significant. The evolution of subsurface defect is mainly driven by the material deformation energy, which results in the scale of subsurface defects increase. And the main types of dislocations in the stage are the green Shockley dislocation and the pink ladder dislocation, as shown in Figs. 9a, b and 10a, b. The interaction between the SFT2 and Shockley dislocation nearby makes pink stair-rod dislocation turn into green Shackley dislocation in the area far away indenter action. Meanwhile, the SFT2 is transformed into a smaller defect which is SFT3, as shown in Figs. 9c and 10c. With the continuous lifting of the indenter, the bonding and adsorbing effect exerted by the indenter on the specimen increases gradually. Accompany with the deformation energy release, the size and types of dislocations in subsurface increase. And more perfect dislocations, Hirh dislocations, and unknown dislocations are formed, as shown in Figs. 9d and 10d. In later stage of unloading process, the material deformation energy is basically released, and the evolution of subsurface defects is dominated by the adsorption from the indenter. Therefore, the subsurface defects are annihilated rapidly, and the scale of subsurface defects decreases rapidly. And a typical Hirsch dislocation is formed in the direction of the indenter upward, which is shown in Figs. 9e, f and 10e, f. Finally, the scale of subsurface defects decreases considerably, and some typical subsurface defects, such as SFT and atomic clusters, are gradually disappeared. From the above analysis, it can be seen that secondary processing (nano-indentation) can restore the typical internal defects formed in nano-cutting, and the subsurface damage becomes more serious in the secondary processing area. These characteristics of subsurface defects will affect the mechanical properties of materials. Hence, it is necessary to study the effect of subsurface defects on the mechanical properties of material.

**Fig. 11:**
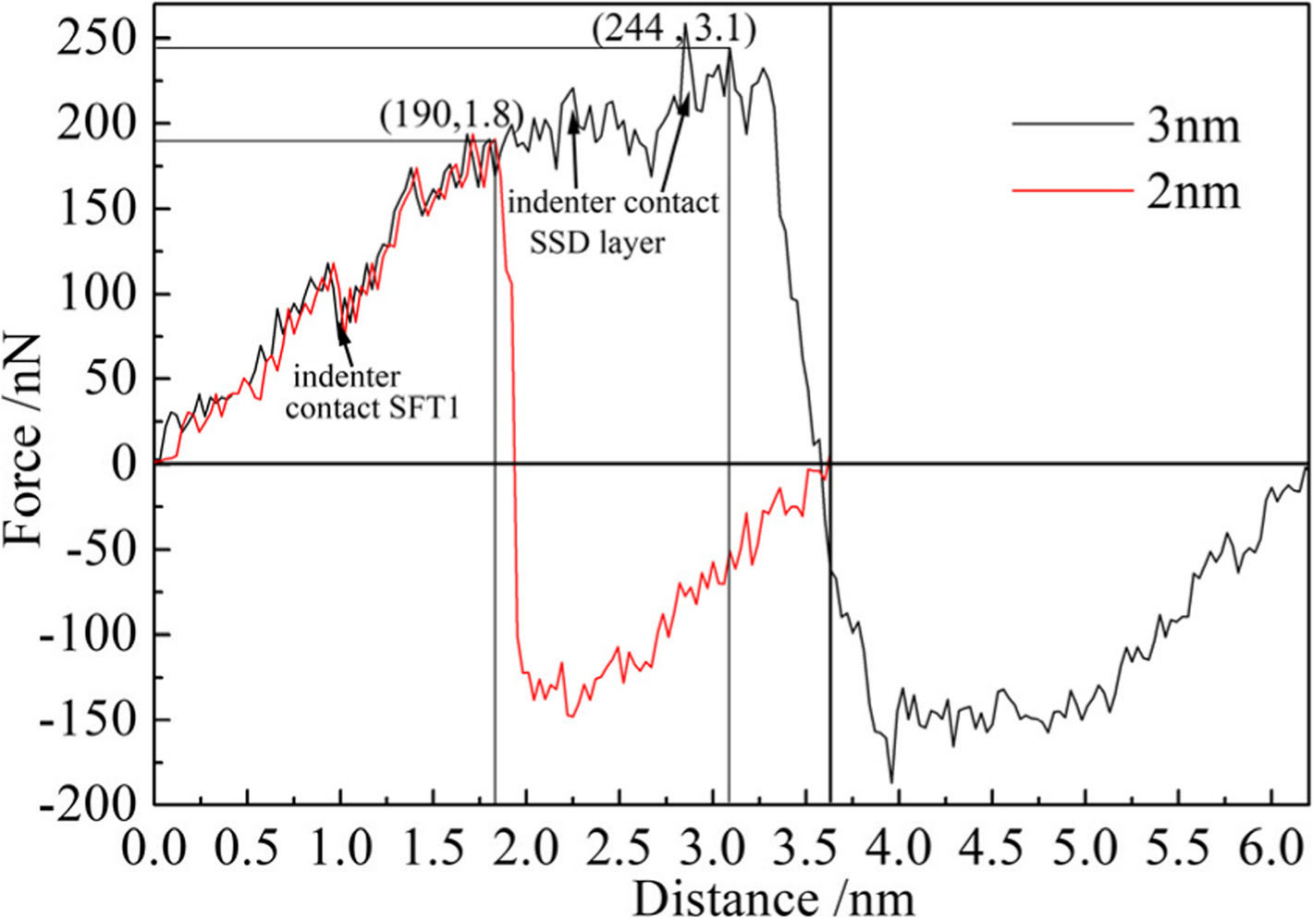
Load-displacement curve of nano-indentation on the machining-induced specimen (color online). The maximum indentation depth of the black curve and the red curve respectively is 3 nm and 2 nm

Mechanical properties of materials can be calculated by load-displacement curve, such as hardness, elastic modulus, and yield strength. In this study, hardness and Young’s modulus of single crystal copper were investigated. The variation of load on the indenter was monitored during the nano-indentation process, and the load-displacement curve of the nano-indentation process was drawn, as shown in Fig. 11. The max indentation depth of the red and black curves respectively is 2 nm and 3 nm, in which both loading and unloading processes are included. The upward direction of load is defined as positive direction, so the load-displacement curves are all above the zero line in the process of loading, while the load on the indenter changes from positive to negative during unloading. The elasticity restore of deformed matrix material exerts an upward force on the indenter. Therefore, in order to keep the indenter raising in uniform speed, a downward force (positive) is required. With the gradual recovery of deformation, the force gradually decreases until it disappears. Then the force applied on the indenter becomes negative, and the absolute value of the force first increases and then decreases. From the graph, it can be seen that the specimen is in the stage of elastic deformation during loading process, and the load increases in proportion to the displacement. When the displacement of the indenter is 1 nm, the load on the indenter is fluctuated dramatically, as indicated by the arrow on the left side of Fig. 11. This is because that the indenter is pressed down to the SFT1 shown in Fig. 7, which results in the greater impediment to the indenter. When the indenter displacement is in the range of 2 to 3 nm, the fluctuation amplitude of the load increases. This is because the indenter is pressed down to the SSD layer, and the impediment of the indenter to the subsurface defect in the specimen is more significant, so the fluctuation amplitude of the load increases significantly.

According to Oliver-Pharr Method [31], nano-indentation hardness can be defined as indentation load divided by the contact area between indenter and specimen, which is calculated by Eq. 7.

7 $$ H={F}_{\mathrm{max}}/{A}_c $$

In which, *F*_max_ is the peak value of the load. *A*_*c*_ is projected contact area for indenter and specimen, which can be obtained by

8 $$ {A}_c=\pi {r}^2 $$

where *r* is contact radius for indenter. Because the indenter is hemispherical in shape, *r* is approximately equal to indentation depth.

The elastic deformation of single crystal copper material is caused by the load exerted from indenter. Based on the previous load-displacement curve, Young’s modulus (*E*) of single crystal copper can be calculated by Eq. 9.

9 $$ E=\frac{\sigma }{\varepsilon }=\frac{F/S}{DL/L}=\frac{F/\pi {r}^2}{DL/L} $$

where *F* is applied load, *S* is contact area, *r* is projection radius of indenter, DL is material deformation along loading direction, which is equal to indentation depth, and *L* is the total length of material along loading direction. In this study, *L* is equal to 30 nm.

According to the Oliver-Pharr method and load-displacement data, nano-indentation hardness and Young’s elastic modulus of single crystal copper materials with subsurface defects can be calculated based on Eqs. 7, 8, 9. Table 3 shows the applied load acting on the specimens by indenter with different indentation depths.

**Table 3:** Applied load on spacemen with different indentation depth

Maximum depth 2 nm	Indentation depth	0.5 nm	1 nm	1.5 nm	Maximum load *F*_max_ (depth)
	Applied load (nN)	45.79	83.56	154.70	190.67 (1.8 nm)
Maximum depth 3 nm	Indentation depth	0.5 nm	1 nm	1.5 nm	244.66 (3.1 nm)
	Applied load (nN)	46.18	87.71	161.49	
	Indentation depth	2 nm	2.5 nm	3 nm	
	Applied load (nN)	199.40	213.29	234.61	

The nano-indentation hardness of single crystal copper can be calculated by Eqs. 7 and 8 combining the data in Table 3. When the maximum depth is 2 nm, *F*_max_ = 190.67 nN and from Fig. 11 *r* = 2.75 nm. The calculated value of nano-indentation hardness H2 is 8.029 GPa. When the maximum depth is 3 nm, *F*_max_ = 244.66 nN and *r* = 3 nm. The calculated value of nano-indentation hardness H3 is 8.675 GPa, which is slightly larger than it obtained at indentation depth of 2 nm. It is because the indenter is pressed down to the subsurface defects area at indentation depth of 3 nm, and the deformation resistance of the subsurface defects increases. Therefore, the hardness of the single crystal copper increased. It can be concluded from the result that the subsurface defects make the machined surface much harder, which is work-hardening phenomenon.

The Young’s modulus *E* of single crystal copper can be calculated by Eq. 9 combining the data in the Table 3. The calculation results are shown in Table 4. It can be noted that the Young’s modulus becomes distinctly higher when the indentation depth is smaller than 1.5 nm. In the initial stage of nano-indentation, the indenter does not contact the defects residual area in subsurface. However, the work-hardening effect makes the specimen material not easily be occurred elastic deformation; therefore, the Young’s modulus of single crystal copper is larger in the initial stage of indentation. The value of Young’s modulus is 119.4 GPa when indentation depth is 2 nm, which is almost the same with Zhang’s research (120.4 GPa) [14]. With the increase of indentation depth, the Young’s elastic modulus of single crystal copper specimens decreases gradually, and the ability of materials to resist elastic deformation is weakened. It is due to the permanent elastic deformation is derived from the dislocation motion and its interaction with subsurface defects. It has been revealed that the nano-cutting-induced subsurface defects will affect the physical and mechanical properties of single crystal copper materials, which is also applicable to other FCC materials. The existence of subsurface defects will enhance the hardness of machined surface and weaken the ability of material to resist elastic deformation, in which the mutual evolution between dislocations and subsurface defects plays an important role. Therefore, it is very important to predict the thickness of subsurface deformation layer and study the surface properties for nano-fabrication.

**Table 4:** Young’s modulus of single crystal copper during nano-indentation with subsurface defects

Maximum depth 2 nm	Indentation depth	0.5 nm	1 nm	1.5 nm	Maximum load depth (depth)
	Young’s modulus *E* (GPa)	160.69	160.54	147.46	134.38 (1.8 nm)
Maximum depth 3 nm	Indentation depth	0.5 nm	1 nm	1.5 nm	86.57 (3.1 nm)
	Young’s modulus *E* (GPa)	162.06	168.51	153.34	
	Indentation depth	2 nm	2.5 nm	3 nm	
	Young’s modulus *E* (GPa)	119.40	93.66	83.02	

## Conclusions

The subsurface defects stable exist in workpiece after nano-cutting can affect the mechanical properties, which is critical to the service performance and life of nano-structures. The previous studies are mainly based on perfect crystal materials or artificial constructing hypothetical defects, which is far from the actual subsurface defects. In this paper, molecular dynamics simulation of nano-cutting is performed to construct the specimen model with subsurface defects. Based on the built MD model, nano-indentation simulation is carried out to study the influence of machining-induced subsurface defects on the physical and mechanical properties of single crystal copper materials. The interaction mechanism between dislocation and complex defects during nano-indentation is studied. The nano-indentation hardness and Young’s elastic modulus of single crystal copper materials are calculated. Based on the above analysis, some interesting conclusions can be drawn as follows.

1. The dislocation nucleation and expansion in workpiece subsurface are driven by the extrusion and shearing action of cutting tool during the nano-cutting process, which results in the fluctuation of cutting force. After nano-cutting, there are stable defect structure residues in the subsurface of workpiece, such as vacancy defects, stacking faults, atomic clusters, SFT, and stair-rod dislocations, which together constitute the subsurface defect layer of workpiece.
2. The existence of subsurface defects affects the nucleation and expansion of dislocations during nano-indentation process. Some stable defects directly below the indenter, such as V-shaped dislocation and SFT1, are annihilated after indentation. And SFT2 far from the indentation region is transformed into a smaller one. Secondary processing of nano-indentation can restore typical internal defects of the workpiece, but the subsurface defects in the secondary processing area are aggravated.
3. The nano-indentation hardness of specimens increases with the introduction of subsurface defects, which results in the formation of work-hardening effect. The Young’s modulus of single crystal copper is larger in the initial stage of indentation and gradually decreases with the increase of indentation depth. The existence of subsurface defects can weaken the ability of material to resist elastic deformation, in which the mutual evolution between dislocations and subsurface defects plays an important role.

## Data Availability

The conclusions made in this manuscript are based on the data which are all presented and shown in this paper.

## Abbreviations

MD: Molecular dynamics
SSD: Subsurface defects
SFT: Stacking fault tetrahedral
PBC: Periodic boundary condition
LAMMPS: Large-scale atomic/molecular massively parallel simulator
EAM: Embedded-atom method
CSP: Centro-symmetry parameter
FCC: Face center cubic
CNA: Common neighbor analysis
HCP: Close-packed hexagonal
BCC: Body centered cubic
ICO: Icosohedral
DXA: Dislocation extract algorithm
